# Exploring challenges of health system preparedness for communicable diseases in Arbaeen mass gathering: a qualitative study

**DOI:** 10.12688/f1000research.15290.1

**Published:** 2018-09-11

**Authors:** Arezou Karampourian, Zohreh Ghomian, Davoud Khorasani-Zavareh

**Affiliations:** 1 Department of Health in Disasters and Emergencies, School of Public Health and Safety, Shahid Beheshti University of Medical Sciences, Tehran, 198353-5511, Iran; 2 Safety Promotion and Injury Prevention Research Center, Shahid Beheshti University of Medical Sciences, Tehran, 198353-5511, Iran; 3 Department of Clinical Sciences and Education, Karolinska Institute, Stockholm, 202100-2973, Sweden

**Keywords:** Preparedness, Health system, Infectious diseases, Religious mass gatherings

## Abstract

**Background: **Infectious diseases are common problems in mass gatherings, especially when there is a lack of health system preparedness. Since Iran is one of the most important countries on the walking path of Arbaeen and has a vital role in providing health services to pilgrims, the experiences of health challenges by participants is of key importance. The aim of this study is to explore stakeholders’ experiences on the health system's preparedness and challenges, and to provide suggestions for preventing infectious diseases during the Arbaeen mass gathering.

**Methods: **A qualitative research method was used with a conventional content analysis approach. The number of participants was 17, including 13 executive managers and 4 health policymakers who entered the study among participants. Semi-structured interviews were used to generate the data. Interviews were analyzed by means of content analysis after face-to-face interviews.

**Results:** Data analysis resulted in the extraction of four main themes and 11 sub-themes. Health infrastructure defects in Iraq has three sub-themes (health abandonment in Iraq, the weaknesses in health culture and problems related to the health system); poor control of the causative factors of infectious diseases has three sub-themes (the underlying factors of the prevalence of contagious diseases, health system response to communicable diseases and ignoring the risks of the Arbaeen ceremony); the low perception of risk in pilgrims has three sub-themes (lack of awareness in pilgrims, fatalism in pilgrims and unhygienic belief in pilgrims); and the ineffectiveness of health education has two sub-themes (training shortage in the targeted group and educational content problems) that shows participant’s experiences of the health system's challenges for coping with infectious diseases during the Arbaeen ceremony.

**Conclusion: **Pilgrim-based training, planning and controlling other challenges may change these threats to opportunities and improve the health of participants of the mass gathering of Arbaeen in the region.

## Introduction

According to the definition of the World Health Organization (WHO), any structured or spontaneous event leading to a certain number of people gathering in a particular site, for a specific aim in a determined period, putting pressure on the response resources and social programs, is called a mass gathering. Mass gatherings are divided into different types based on their purpose. The expansion of interconnectivity between societies and increases in the number of national and international events in communities has led to an increase in the number of mass gatherings, which, despite the benefits like cultural exchange, have health challenges such as infectious disease transmission and, should therefore be considered by health planners ^1^.

One of the health challenges of mass gatherings is the prevalence of infectious diseases and the outbreak of diseases, which, along with, Complicated health needs of participants increases the health burden on the host country. The public health system can be under severe pressure, even with advanced equipment and the proper resources for prevention and control of infectious diseases ^2– 4^. Various factors, such as the type and location of gathering, the number of participants and the lack of access to health facilities, can affect the incidence of infectious diseases in mass gatherings. Planners must therefore pay attention to these factors in preparation ^1, 5– 7^. Since a mass gathering is a collection of many people together in one particular site, the possibility of infectious disease transmission due to the high population density always exists. Studies on mass gatherings such as Hajj, Ashura day in Karbala, and Kumbh Mela and Sabarimala in India show the prevalence of infectious diseases in these ceremonies ^8– 13^.

The dates of religious ceremonies like Hajj and Arbaeen are decided using the lunar calendar (the Islamic or Hijri calendar) which is not only shorter than the Gregorian calendar, meaning that these events occur 10 days earlier each year and can synchronize with different seasons and season-associated diseases. Accordingly, planners and policy makers of public health are faced with changing goals, requiring health system preparedness ^14, 15^. One of the world’s largest religious gatherings is the Arbaeen ceremony, which happens on the 40 ^th^ day after the anniversary of Imam Hussein’s martyrdom, the third Shiite Imam. In the Ashura event, pilgrims walk to Karbala, south of Baghdad. Based on the statistics of 2017, the number of Iranian pilgrims taking part in the Arbaeen ceremony was 2,320,000 ^16^.

Iran is a neighboring country of Iraq and shares a common land border with it. Pilgrims from other neighboring countries of Iran, such as Afghanistan and Pakistan, cross Iran to reach Iraq and the Arbaeen ceremony. On the basis of the Mutual Memorandum of Cooperation between the two countries of Iran and Iraq, Iran is committed to providing health services to other pilgrims in addition to Iranian pilgrims ^4^. Therefore, it is necessary to have a plan for preparedness in dealing with infectious diseases during Arbaeen ceremony.

If a mass gathering is not carefully managed, it can lead to the distribution of infectious diseases. In mass gatherings, infectious diseases are threats to global health security and even the political security of countries. Therefore, planning, communication and public health supervision are important in these religious ceremonies ^1, 8^. Mass gatherings are different from structured disasters so that in case of occurrence, many people will be affected ^17^. Since the Arbaeen ceremony is held with the presence of many pilgrims from many different countries and, like the Hajj pilgrimage, is based on the lunar calendar, and it is held in Iraq, there is the possibility of the occurrence and transmission of infectious diseases. It is therefore essential to be prepared to control and prevent these diseases. Dealing with infectious diseases in Arbaeen is considered a challenge for policy makers ^10^. According to Arbaeen's social and cultural context, it seems essential to take a deeper look in this field. On the other hand, there is relatively little knowledge about the Arbaeen ceremony; therefore, a qualitative method for clarifying the concept and challenges of health system preparedness in Arbaeen ceremony is necessary. The aim of this study was to explore challenges of health system preparedness for communicable diseases in Arbaeen ceremony.

## Methods

### Approach

We collected data from June 2017 to March 2018. Since the study attempted to explore preparedness challenges of health systems, a qualitative research method with the approach of conventional content analysis was used ^18^. The health system's challenge in Arbaeen is multidimensional, Owing to the cultural difference between Iran and Iraq, the challenges faced by the health system during the Arbaeen pilgrimage should be investigated in both countries. Indeed, the cultural practices of the participants, especially those surrounding health, differs during the Arbaeen ceremony. Therefore, a qualitative research method, with the aim of describing phenomena, providing new knowledge, insight and a practical health guide, is the method used in this study ^18, 19^.

### Setting

The study was conducted using in-depth interviews, based on stakeholder’s experiences in Iraq-Iran land terminals (Mehran, Shalamche, and Chazaba) and also in health care posts in Iraq. The interviews were conducted with health care providers and policy makers, as well as pilgrims in the Arbaeen ceremony.

### Participant selection

Participants were chosen among executive managers and policymakers of the Ministry of Health and Medical Education, medical training and treatment and other related organizations, including the Red Crescent organization, Mobilizing the Medical Society, the Hajj and Pilgrimage Organization, medical universities in the border cities and the Social Security Organization. In total, 17 participants, consisting of 13 executive managers and 4 health policymakers, were selected in this study through purposeful sampling with the aim of exploring challenges of health system preparedness for communicable diseases in the Arbaeen ceremony. The existence of practical experience in planning or participate in Arbaeen ceremony and the ability to communicate and willingness to participate were inclusion criteria in the research. We recruited participants either by phone call or by approaching them in person.

"Maximum variety sampling" was used to explore the experiences of the participants in those selected so that they were chosen from the Ministry of Health and Medical Education, medical training and treatment and other related organizations, including the Red Crescent organization, Mobilizing the Medical Society, the Hajj and Pilgrimage Organization, medical universities in the border cities and the Social Security Organization, with different experiences of work, education and gender. Inclusion criteria included the existence of practical experience in planning or participate in Arbaeen ceremony, the ability to communicate and willingness to participate in the research ( Table 1).

**Table 1.** Demographic characteristics of participants of the study at Arbaeen ceremony in 2017.

Variables		N (%)
Participants	Executive managers	13 (76.47)
	Policy makers	4 (23.53)
Age, years	30–40	8 (47.05)
	41–50	6 (35.3)
	51–60	3 (17.65)
Sex	Male	15 (88.24)
	Female	2 (11.76)
Job experience, years	Less than 10	2 (11.76)
	11–20	7 (41.18)
	21–30	6 (35.3)
	Over 30	2 (11.76)
Number of visits to Arbaeen	0	3 (17.65)
	1–5	9 (52.94)
	6–10	5 (29.41)

### Data gathering

The study was done through face-to-face interviews followed by telephone interviews for concept saturation. The data was collected using audio recorders with permission from the participants. AK conducted the interviews, AK and DKZ transcribed the data. AK and DKZ and ZGH coded the data. DKZ and ZGH performed rigor. Initially the first two interviews were conducted in a non-structured format, with the following 15 interviews being semi-structured. Open questions used to generate the data were developed by experienced and/or knowledgeable policy makers and health care providers. The individual’s experiences and beliefs were used without considering their specialty ^20^. Interviews were continued until data and concept saturation were reached ^21^. The interview duration was between 35 and 95 minutes, based on the tolerance, amount of information and desire of the participants. Interviews were performed individually and based on participants' willingness in terms of time and site.

Firstly, interview questions began with the following general questions based on the participants' level and the main questions of the research: "How was your organization preparedness plan to deal with infectious diseases in Arbaeen ceremony?"; "Please express your experiences of related challenges in infectious diseases in Arbaeen ceremony"; "What problems were in your preparedness plan?"; “What problems did you face in the vaccination program of the health team and pilgrims in Arbaeen ceremony?”; and “What is your offer to pilgrims for a safe pilgrimage?" Following this, exploratory questions were gradually used to clarify the concept and deepen the interview process: e.g. “Please explain further what you mean?” and “Why?” At first, the interviews were transcribed verbatim and then typed up using Microsoft Word Office.

### Data analysis

The data were analyzed through a conventional content analysis method ^18^. First, the main researcher converted the interviews to written texts. The digital files were listened to several times and the texts were read repeatedly. Next, the meaning units were determined based on the aim and the question of the research. Meaning units were a collection of words and sentences that were related to each other in terms of content and were grouped together. Meaning units reached the level of abstraction and conceptualization and were coded considering the research question. The key points and subjects were extracted as open codes. These codes have been put under the broader headings based on the present similarities and differences; in other words, the data were reduced in order to describe the phenomenon and gain a better understanding, and this abstraction process continued until concept extraction ^18^. Data first emerged as meaning units, then condensed meaning units, codes, sub-themes and finally themes.

### Ethical considerations

The study was approved by Ethics Committee of Shahid Beheshti University of Medical Sciences on 10/08/2017, No. IR.SBMU.RETECH.REC.1396.349. The interviews were conducted and recorded with participants' consent. Written or verbal consent was taken from participants to participate in the study. Verbal consent was only taken for telephone interviews; this was due to the distance between interviewer and interviewee. Anonymity, confidentiality and the right of resignation were informed to participants and considered during the study. The interview time was set according to participants' willingness.

### Rigor

The researchers used the trustworthiness criteria recommended by Guba and Lincoln to establish rigor ^22^. All authors were engaged in the environment and field of research. In addition, the principle investigator always had suitable involvement with the participants for in-depth interviews. Credibility was established by the prolonged engagement of researchers with participants. Researcher triangulation was also used to verify the accuracy of the coding process. The research team also retained raw data, codes, and themes for control of the reliability. At the same time, sampling was carried out with maximum diversity in order to provide triangulation by means of credibility and confirmability. A detailed description of the method was used to establish transferability. Engaging participants in the research increases the interaction between researchers and participants and then the credibility ^22^. The research supervisor monitored the data collection and data analysis process. It is necessary to mention that the research team participated in the Arbaeen ceremony as pilgrim and conducted field notes. Also, member- and peer-checking were used to ensure credibility. Therefore, many interviews with related topics were sent to some external expert reviewers and participants (policymakers of the Ministry of Health and Medical Education, medical training and treatment and the Red Crescent organization) to be checked, and they were requested to assess the degree of relevance between the findings and raw data. Moreover, transferability was established by sampling with maximum variation from various centers, including the Ministry of Health and Medical Education and other peer organizations including the Red Cross organization, the Medical Community Mobilization, the Hajj and Pilgrimage Organization, the country's medical universities in the border cities and the Social Security Organization with different experiences of work, education and gender. A completed SRQR checklist is available in Supplementary File 1.

## Results

### Themes and sub-themes

The mean age of participants was 43 and the mean time of work experience was 18 years ( Table 1). Overall, 1125 original codes were extracted and after integration using conventional concept analysis, four original themes consisting of 11 sub-themes were identified. The theme of “health infrastructure defection in Iraq” had three sub-themes: “health abandonment in Iraq”, “the weakness of health culture” and “problems related to health system”. The theme of “poor control of factors effective in infectious diseases” had three sub-themes: the “underlying factors in prevalence of contagious diseases”, “health system response to communicable diseases” and “ignoring the risks of the Arbaeen ceremony”. The theme of “low perception of risk in pilgrims” had three sub-themes: “lack of awareness in pilgrims”, “fatalism in pilgrims” and “unhygienic belief in pilgrims”. The theme of “ineffectiveness of health education” had two sub-themes: “training shortage in the targeted group” and “educational content problems” ( Table 2).

**Table 2.** The inductive process of abstraction of codes and categories in challenges of health system preparedness for communicable diseases in the Arbaeen ceremony.

Themes	Sub-themes	Initial codes
Health infrastructure defection in Iraq	Health abandonment in Iraq	Lack of effective supervision in the field of health
		Lack of health surveillance on food supply
		The weak implementation of health regulations
	The weakness of health culture	Disregard for the implementation of individual and public health
		Lack of health belief
	Problems related to health system	Poor health background
		Problems in collecting sewage and waste disposal
Poor control of the effective factors on infectious diseases	The underlying factors in prevalence of contagious diseases	Lack of personal and public health facilities
		Inadequate space to settle
		Air pollution
		Diversity and population density
	Health system response to communicable diseases	The probability of an outbreak of endemic and non-native diseases
		The ineffectiveness of the health system in screening
		The impossibility of requirement flu vaccination
		The inability of the system to provide services in an epidemic
		The probability of a bioterrorism agent coming to Iran
		Lack of attention to emerging and reversible illnesses
	Ignoring the risks of the Arbaeen ceremony	Ignoring gathering of people with different cultures and ethnicities
		Inattention to the animated crowd of pilgrims
		Omitting the existence of various hazards during the trip
Low perception of risk in pilgrims	Lack of awareness in pilgrims	Lack of knowledge about personal and public health
		Lack of knowledge about proper nutrition
		Refuse to take medication
	Fatalism in pilgrims	Belief on no need for travel planning for Arbaeen ceremony
		The pilgrims believe that they will not be ill and safe from the hazards
	Unhygienic belief in pilgrims	The prevalence of self-treatment in pilgrims
		Lack of attention to health and health advice
Ineffective health education	Training shortage in the targeted group	Lack of training in pilgrims
		Lack of training in policy makers and executives of Arbaeen ceremony
		Inadequate training of volunteers
		Late pilgrimage training
	Educational content problems	Low proportion of training with the need of pilgrims
		Lack of training in food hygiene
		Inadequate training of individual and public health

According to the findings of this study, the main theme is pilgrim-based education. It seems that the biggest issue with the prevention of communicable diseases in the Arbaeen ceremony is pilgrim-based education. Educating pilgrims can directly help people to create or reform health infrastructures in Iraq. Education can also help to identify health risks and respond to them by identifying effective factors in combating infectious diseases. The training of health instructors and guidelines from people who are trusted and accepted by pilgrims, such as missionaries and religious leaders, can have a positive impact on their beliefs. The main point of training is to determine the targeted group both at the level of pilgrims and health directors and consider the training needs of each group in preparing training guidelines.

### Health infrastructure defection in Iraq

***Health abandonment in Iraq.*** Most of the participants believed that the Iraqi health system has been abandoned because foodstuff distribution is not under the supervision of a special organization and does not have a special trustee in the execution and monitoring of health rules or, if there is one, he is inconspicuous. The existence of a trustee or supervisor in the health system can prevent the delivery and distribution of unsafe food and reduce the prevalence of infectious diseases. Unhealthy foodstuff preparation, production and distribution, and a lack of food evaluation and supervision system can lead to gastroenteric disease. Based on the participants’ experiences, the lack of a health system trustee means system weakness.

The following quotation is an example of the above: *"Sometimes donations are prepared in an unhealthy manner and so lead to acute digestive problems… The health system is weak in Iraq because health rules are not enforced and there is no supervision for these centers…"* (Executive manager, male, 30–40 years, 11–20 years job experience).

**The weakness of health culture**. According to the participants' point of views, observation of unhealthy behaviors, such as neglect of the individual and public health standards, and the existence of cultural differences between Iran and Iraq, are considered by pilgrims to bring about an unsafe culture. Policy makers and executives should be familiar with the kind of health culture in Iraq so that they can develop a program to prevent contagious diseases. In Iranian culture, the non-use of spoons and forks is considered as lack of health belief and neglect of health, whereas in Iraq, eating food with the hands is part of the food culture. Iran's health system can help train the Iraqi people in sanitary practices alongside Iranian pilgrims.

The following quotation is an example of the above: *"Some food providers don’t meet health … The culture of using spoons and forks for food serving is different in Iran and Iraq… one of the cultural works which we can do in Iraq is health education…"* (Executive manager, male, 41–50 years, 11–20 years job experience).

***Problems related to health system.*** Most participants acknowledged that despite annual health care improvements in Iraq, there are also some shortages in this field due to the lack of a long history of a health service system. Health system weakness, incomplete health service implementation and insufficient supervision of environment health are indicative of the weakness of the health infrastructure; this has made it impossible to provide environmental health, sanitation and waste disposal.

The following quotation is an example of the above: *“Health background in Iraq was poor… There were no waste bins and waste sanitation there… failure to implement health services has led to failure in meeting health conditions … there was no health system supervision and waste collection system… of course every year its gets better than the previous year … "* (Executive manager, female, 41–50 years, 21–30 years job experience).

### Poor control of the effective factors on infectious diseases

***The underlying factors in prevalence of contagious diseases.*** Most participants have identified underlying factors of infectious diseases as one of the challenges affecting the preparedness of the health system in dealing with infectious diseases. Various factors, such as population density and diversity, not paying attention to the principles of personal and general health, for example through a lack of health facilities, weather conditions during the trip and changes in nutrition can cause the spread of infectious diseases. Identifying these factors helps pilgrims and planners to prevent infectious diseases.

The following quotation is an example of the above: *"There were few toilets or no healthy facilities. Somehow, pilgrims would have to sleep in the desert or in a limited space with a lot of people… Congestion of pilgrims from different countries increases the risk of spreading infectious diseases…”* (Executive manager, male, 51–60 years, Over 30 year job experience).

***Health system response to communicable diseases.*** On the basis of the view of participants, mass population movements from different countries and their gathering with different population diversity can transfer and spread endemic diseases and also emerging diseases such as plague and anthrax. There is also the possibility of bioterrorism events occurring during the Arbaeen ceremony. The health system needs facilities such as equipped laboratories to diagnose and treat infectious diseases in a timely manner. The inaccessibility or lack of experimental equipment for disease identification leads to the failure of timely diagnosis of diseases, a lack of disease control and finally the incidence of epidemics. Syndromic surveillance system can be used to diagnose infectious diseases. There is a need to correctly pass the treatment period of infectious diseases in order to prevent epidemics, although drug shortages can affect treatment completion and is one of the causes of epidemics. Vaccination also helps to prevent infectious diseases, but the requirement of pilgrims to vaccinate is always up to the host country and since vaccination is not one of Iraq's priorities, the Ministry of Health and Medical Education can only advise pilgrims to vaccinate.

The following quotation is an example of the above: *"There is the probability of spread of local and new-appeared diseases and even bioterrorism due to the gathering of pilgrims from different countries… some diseases can't be diagnosed due to lack of facilities but syndromic surveillance system can be used. …the large number of pilgrims and lack of medication have led to not having complete course of antibiotic treatment… The need for vaccination is one of the requirements of the host country".* (Policy makers, male, 41–50 years, 21–30 years job experience).

***Ignoring the risks of the Arbaeen ceremony.*** The Arbaeen pilgrimage has special features that distinguish it from other mass gatherings. Participation of different peoples with a diverse range of socio-demographic statuses, cultures and nationalities makes this distinction. The Arbaeen pilgrimage has some specific hazards like other trips that are sometimes neglected. The population of Arbaeen pilgrims and its time of year are changeable. Financial management of travel expenses at the ceremony is carried out by volunteers. Considering these features is essential for the readiness program. In the opinion of the majority of participants, one of the challenges of the health system is the lack of attention to the risks of Arbaeen ceremony and the lack of planning based on these features.

The following quotation is an example of the above: *"Arbaeen is a new phenomenon that children and adults, men and women with different cultures and ethnicities take part in the ceremony … Even with the knowledge of the dangers of the route, people attend Arbaeen ceremony… Arbaeen is a spontaneous and popular event and doesn’t cost much … the population is moving and the time of the ceremony changes every year …"* (Executive manager, male, 30–40 years, 21–30 years job experience).

### Low perception of risk in pilgrims

***Lack of awareness in pilgrims.*** One of the challenges in the view of the participants, especially executives, is the pilgrims' low awareness of health hazards, such as not using personal hygiene products and non-compliance with health standards. Unhealthy and dangerous practices, such as unsanitary food consumption, are not understood by pilgrims, and this low awareness and inadequate knowledge of the risks are the causes of infectious diseases in the pilgrims. Respect for personal and public health, such as hand washing, providing food from health food centers and avoiding overeating, is effective in preventing digestive diseases.

The following quotation is an example of the above: *"Some pilgrims do not wash their hands or do not use personal hygiene products …Or they do not get foods from healthy centers … overeating and then digestive problems are one of the pilgrims' problems … the other problem is not being familiar with Arabic language …"* (Executive manager, female, 30–40 years, less than 10 years job experience).

***Fatalism in pilgrims.*** Participants believed that one of the health challenges is belief in destiny and fatalism; so that most pilgrims who participate in the Arbaeen walking ceremony, based on their belief in destiny, have a relatively low understanding of the dangers and diseases, and begin their trip merely confident in Allah and without any plan for dealing with infectious diseases, such as vaccination, and continue the way using food and drinks that are often unhealthy.

The following quotation is an example of the above: *"It's enough to decide to travel and you do not need to have a special plan … If you think openly, you won’t get sick, and nothing bad will happen…"* (Policy makers, male, 41–50 years, 21–30 year job experience).

***Unhygienic beliefs in pilgrims.*** Based on the participants’ experiences, low perception of danger in pilgrims is sometimes seen as insanitary beliefs in preventing medication consumption and disregarding hygiene recommendations. Believing in the use of medication during illness, preventing self-curing and trying to abide by hygiene recommendations prevents infectious diseases.

The following quotation is an example of the above: *"Sometimes we see pilgrims using traditional treatments instead of antibiotics consumption… they don’t pay attention to hygiene recommendations such as masking and not using suspicious food…"* (Executive manager, male, 30–40 years, 11–20 year job experience).

### Ineffectiveness of health education

***Training shortage in the targeted group.*** Based on stakeholders' views, one of the present challenges is the quantitative and qualitative shortage in the stakeholder’s training level. This means that providing a personal and public health plan should cover all stakeholders, including executives, policymakers and volunteers in the Arbaeen ceremony, and be considered with respect to each participant. Furthermore, the timing of training is also important and should be before the days of Arbaeen ceremony in order to have a greater effect on the individuals' knowledge. Indeed, training courses should be held separately for each of the groups, pilgrims, executive managers, and policymakers, and at a proper time before Arbaeen.

The following quotation is an example of the above: *“Training should not exactly be in the days of Arbaeen. Personal and public health training must be held several months before Arbaeen… the training is not only for pilgrims, but also anyone involved in Arbaeen ceremony. Everyone should be trained from pilgrims to policymakers…"* (Policy maker, male, 41–50 years, 21–30 year job experience).

***Educational content problems.*** Another problem was the provision of educational content. Most of the participants believed that training should be fitted with pilgrims' needs and respond to the problems of pilgrims. Pilgrims should be divided into different groups based on the level of education, their problems and illnesses, and individual and general education should be planned accordingly. Participants also believed that training in the area of personal, general and nutritional health should be provided more comprehensively.

The following quotation is an example of the above: *"We should divide pilgrims to diverse groups and send targeted training messages to each group, not the same training for everyone…the benefactors should be trained…pilgrims should have more training…"* (Policy maker, male, 41–50 years, 21–30 year job experience).

## Discussion

The aim of this study was to explore the challenges of health system preparedness for infectious diseases in the Arbaeen ceremony as the first qualitative study in Iran. The most important findings of the study are the ineffectiveness of health training, the low perception of risk in pilgrims, poor control of the causative factors of infectious diseases, and deficient and defective health infrastructure in Iraq. Based on the views of the majority of participants, pilgrim-based training is the most effective factor in health system readiness in dealing with infectious diseases in the Arbaeen ceremony.

Ineffectiveness of health training is one of the challenges of health system preparedness. One of the plans which should be considered to ensure Arbaeen ceremony preparedness is health training. Educational planning must be done before holding the Arbaeen ceremony, with consideration of the training content and targeted groups. Indeed, according to the needs of the targeted group, training must be given to pilgrims, executives and volunteer treatment teams, and the training content for pilgrims should be different to that of executives and policy makers. Past conducted studies of religious gatherings such as the Hajj in Saudi Arabia and Ashura Day in Iraq, as well as other mass gatherings, such as the Tamworth Country Music Festival, Australia, indicate the reality that a crowd of people from diverse nations and cultures is a source of infectious disease; disease transmission is one of the most important challenges of public health in these kinds of events, so using training strategies in relation to hand washing, masking and vaccination is an important factor in preventing the diseases and health improvement ^8, 14, 16, 23– 33^. Since religious ceremonies are rooted in people's beliefs and have great popularity among people, people-centered education therefore has great potential to reduce the gap between knowledge and practice in pilgrims, empowering individuals and enhancing their ability to deal with health threats. This goal is achieved by identifying accurate and targeted needs, developing relevant content and through educational planning ^34^. In this study, like other studies on mass gatherings, a personal health training plan, such as the importance of hand washing, using healthy food and masking, should be included in the pilgrims' training plan, and public health training such as vaccination, environmental health, monitoring donations, cooking and distribution, controlling bioterrorism and establishing mobile toilets should be considered in executives and policymakers' preparedness plan. Regarding the aim of holding the Arbaeen ceremony, which is a popular religious–ideological ceremony, training can be performed in mosques before the ceremony by clergy.

A low understanding of the risks of infectious diseases in pilgrims is one of the other challenges mentioned by the majority of participants, especially policymakers. A lack of risk understanding refers to the inability to identify and respond to dangerous situations. Top documents such as Sendai framework ^35^ have identified that understanding risks is the first priority to decrease the incidence of disasters, and that it needs people-based and vast preventive approaches. Besides this, the Hyogo framework and sustainable development goals emphasize the role of training in increasing risk understanding and decreasing the vulnerability of individuals to hazards ^35– 37^. In this study, one of the health issues was fatalism and a low understanding of risk. A study concerning the beliefs and methods of infection control in Hajj pilgrims residing in Australia also showed that the majority of participants had low understanding of the occurrence of respiratory infections and the need for an influenza vaccine in Hajj, and refused the vaccine, using trust in Allah as an excuse and belief in destiny when dealing with the risk of disease ^38^. Another problem is also the prevalence of self-treatment among pilgrims. In a study aimed at assessing the knowledge, attitude and the performance of Australian pilgrims on using antibiotics in Hajj showed that they did not have proper understanding of using these drugs and used medications arbitrarily, meaning that more training on proper use is needed ^39^. A lack of understanding of health instructions and disregard for public and personal health in any situation can endanger human health. It seems that the fatalism of pilgrims with regards to diseases increases their vulnerability if the understanding of danger is reduced. Islamic instructions state that the person is obliged to preserve his health and life in any location and position, even in holy lands, and to avoid risks ^40^. Given that Arbaeen is a religious gathering, religious leaders have an important position in ceremony implementation and can affect the pilgrims' beliefs and understanding of risks during pilgrimage. The influence of religious leaders on the people's beliefs can provide the opportunity to promote health. We should consider that religious scholars must be trained first and then transfer health instructions and methods of infectious disease control to the people in cultural and religious gatherings such as mosques.

Another problem in this field is the poor control of the effective factors on infectious diseases. One of the most common causes of infectious disease is the epidemiology triangle, which has three components—pathogen, host and environment. The interaction of these agents causes infectious diseases, and considering these factors can help control infectious diseases ^41^. Considering the three factors in Arbaeen is very important. The ‘host’ factor includes different pilgrims with diverse cultures and nationalities. The ‘environment’ factor includes holding the ceremony in Iraq, which, due to the many years of internal and external conflicts, has had little attention paid to its health infrastructure, and also the overall environmental factors effective in the occurrence of infectious diseases. Different studies ^1, 8, 42^ have shown that factors such as crowd size, equipment, climate, the event duration and location, the type of ceremony, and features and behavior of participants affect the occurrence of diseases in mass gatherings, and planners must consider it during preparations. To prevent the occurrence of contagious diseases and their consequences, comprehensive planning, rapid diagnosis and effective management is required ^1, 8, 42^. One of the factors that affects the occurrence of disease is the time of year that the Arbaeen ceremony is held. Arbaeen ceremony is held based on the lunar calendar and so its needs and challenges differ and are dependent on the season that the ceremony is held, so that if the ceremony is held in cold seasons, respiratory diseases are more common and if is held in hot seasons, the majority of infectious diseases are of the digestive system. Additionally, population movement among different countries leads to the transfer of local diseases, so health considerations and cooperation between states are needed.. Policymakers should consider three components—pathogen, host and environment—before the beginning of Arbaeen mass gathering. It is also necessary that the health system is aware of all possible scenarios and the methods for dealing with them. The preparedness plan at the local level includes assessing the risk, resources capacity, equipment, surveillance system and an expert team for providing services to pilgrims.

Health infrastructure defects in Iraq was another challenge to the health system from the participants' perspective. The term ‘health infrastructures’ refers to health facilities and their related factors. The infrastructure includes staff instructions, processes and the development of systematic approaches related to personnel resources and medical support plans ^43^. Various studies indicate that readiness for structured mass gatherings depends on investing in health infrastructures and the size of gatherings, and strengthening infrastructures and post-event coordination of mass gatherings must be continued. The inappropriate location of gatherings, the weakness of facilities and the lack of infrastructure increase the vulnerability of communities ^44, 45^. The remoteness of health facilities and a lack of needed road infrastructure can make medical services and emergency assistance ineffective. Limitation of infrastructure and medical care system, increase the incidence of injuries ^44, 45^. Arbaeen is held in a country that has long been involved with interior and exterior wars; therefore, it seems that due to economic difficulties, it does not have the capacity to support the necessary health infrastructure required by pilgrims. Although according to the participants, the health system in Iraq seems somewhat weak, given that Arbaeen ceremony is of particular popularity among Shia Muslims and is held annually, therefore, some of the activities serving pilgrims and its management during the Arbaeen ceremony is voluntarily conducted. In recent years, numerous health facilities have been constructed on religious places, as well as along the path of pilgrimage using pilgrims' donations. Management of pilgrims’ donations can help to build and maintain health infrastructure in Iraq. With this policy, the Iranian pilgrims will benefit from the Arbaeen ceremony, and the level of health in the region will be improved.

## Limitations and strengths of the study

This study is the first qualitative study on the experiences of Arbaeen, so it provides rich information in this regard; however, since the results have been collected from semi-structured interviews, it is considered subjective. It is recommended that in future studies, by creating a quantitative instrument for examining the challenges and measuring, the subjective concepts can be objectively transformed and analyzed. The present study can be used as a basis for this purpose.

## Conclusion

The ineffectiveness of health training, low perception of risk in pilgrims, poor control of the effective factors on infectious diseases and deficient health infrastructure in Iraq are important challenges of the health system in dealing with contagious diseases in the Arbaeen ceremony from the stakeholder’s perspective. Therefore, pilgrim-based educational planning, along with the control of other challenges, represents an opportunity to improve the health of pilgrims taking part in the Arbaeen ceremony.

## Data availability

The full data for this study are not provided because the transcripts of the interviews contain identifiable and sensitive information. Researchers can apply to access limited de-identified transcripts of interviews from the first author, Arezou Karampourian ( a.karampourian@sbmu.ac.ir) under no strict conditions. Please note that transcripts are only available in Persian.

## Supplementary material

**Supplementary File 1. Completed SRQR checklist.**
