# Supplementary material for: Exploring challenges of health system preparedness for communicable diseases in Arbaeen mass gathering: a qualitative study
Source: F1000Res. 2018 Sep 11;7:1448. [Version 1] doi: 10.12688/f1000research.15290.1 (PMC6234742; doi:10.12688/f1000research.15290.1)
Supplement: Peer review history

# Peer review: Reviewer response for version 1

**DOI:** 10.5256/f1000research.16657.r42004

**Published:** 2019-02-27

**Authors:** Harunor Rashid, Farah Al Ansari

**Affiliations:** 1 National Centre for Immunisation Research and Surveillance of Vaccine Preventable Diseases (NCIRS), Children's Hospital at Westmead, Westmead, NSW, Australia; 2 Sydney Medical School, The University of Sydney, Sydney, Australia

The paper is interesting and adds to the medical literature of Arbaeen Ceremony which has been poorly addressed from a public health research perspective. We hope this paper will serve as a key reference on health aspect of Arbaeen Ceremony in near future. A few minor issues should be addressed before the manuscript is published.

1. The reference list needs to be revised, we note some misplacement error. For example, in the third paragraph of the introduction (p 3), “Based on the statistics of 2017, the number of Iranian pilgrims taking part in the Arbaeen ceremony was 2,320,000”, the reference given for this is number 16. However, ref 16 (Ahmed et al.) was published 8 years ago in 2009. Also in the paragraph that follows, “on the basis of the Mutual Memorandum of Cooperation between the two countries of Iran and Iraq, Iran is committed to providing health services to other pilgrims in addition to Iranian pilgrims”, the reference cited is number 4, which is about Hajj and Umrah and cannot the correct reference. 
2. Repetition is another issue, some information are repeated unnecessarily. The authors may consider language editing.
3. A useful reference that the authors may consider quoting is--Al-Lami F, et al. Pattern of morbidity and mortality in Karbala hospitals during Ashura mass gathering at Karbala, Iraq, 2010 ^1^. 
4. Two other relevant references are—a) Alqahtani AS, et al. Australian Hajj pilgrims' infection control beliefs and practices: Insight with implications for public health approaches ^2^; b) Rahman J, et al. Mass Gatherings and Public Health: Case Studies from the Hajj to Mecca ^3^.

In summary this is a indexable manuscript with minor revision.

We have read this submission. We believe that we have an appropriate level of expertise to confirm that it is of an acceptable scientific standard.

## References

[1] Al-LamiFAl-FatlawiABlolandPNawwarAJetheerAHantooshHRadhiFMohanBAbbasMKamilAKhayattIBaqirH: Pattern of morbidity and mortality in Karbala hospitals during Ashura mass gathering at Karbala, Iraq, 2010. *East Mediterr Health J*.2013;19 Suppl 2:S13-8 24673093

[2] AlqahtaniASSheikhMWileyKHeywoodAE: Australian Hajj pilgrims' infection control beliefs and practices: Insight with implications for public health approaches. *Travel Med Infect Dis*.13(4) : 10.1016/j.tmaid.2015.05.009 329-34 10.1016/j.tmaid.2015.05.009 26028307

[3] RahmanJThuMArshadNVan der PuttenM: Mass Gatherings and Public Health: Case Studies from the Hajj to Mecca. *Ann Glob Health*.83(2) : 10.1016/j.aogh.2016.12.001 386-393 10.1016/j.aogh.2016.12.001 28619416PMC7104005

# Peer review: Reviewer response for version 1

**DOI:** 10.5256/f1000research.16657.r38176

**Published:** 2018-11-12

**Authors:** Abbas Ostadtaghizadeh

**Affiliations:** 1 Department of Disaster and Emergency health, School of Public Health, Tehran University of Medical Sciences, Tehran, Iran

The manuscript is drafted very well. So, I would like to thank all authors for their attempts. Here under, There are some comments for improvement the quality of the manuscript which I hope to be helpful.  

1. In the last paragraph of introduction, there are redundant phrases which can be edited.
2. It is better the manuscript mentions some challenges there were in similar mass gathering in the world.
3. In Methods, the authors mentioned that they have collected data from June 2017 to March 2018, while the study settings have been only health care posts in Iraq and Iran-Iraq borders. I think these posts were active only during Arbaeen period (Oct - Nov. 2017). 
4. The second paragraph of participant selection part is very similar to the first one (redundant). In addition, the correct name of organizations should be mentioned. for example, Iranian Red Crescent Society or Medical Society of Basij.
5. Some English editions might be needed. 
6. In the discussion part, it is better to explain statistically how education and other interventions can influence on controlling of communicable diseases based on past researches. Mentioning the importance of interventions such as hand-washing and .... is not a new matter.
7. It is better to discuss the results respectively as the results mentioned. So, the reader can insure that all results have considered in the discussion part.
8. It is better to explain what pilgrim-based education is and how it controls CD in discussion part and conclusion as well.

I have read this submission. I believe that I have an appropriate level of expertise to confirm that it is of an acceptable scientific standard.

# Peer review: Reviewer response for version 1

**DOI:** 10.5256/f1000research.16657.r38175

**Published:** 2018-10-03

**Authors:** Mahnaz Khatiban

**Affiliations:** 1 Hamadan University of Medical Sciences, Hamadan, Iran; 2 Mother & Child Care Research Center, Dept. of Medical Surgical Nursing, School of Nursing and Midwifery, Tehran University of Medical Sciences, Tehran, Iran

This is an interesting and well-written piece of work.

 Infectious disease prevention strategies in mass gatherings, especially religious or cultural gathering, need to start with such the qualitative study. Having a predetermined program, implementation, evaluation, and upgrade of the strategies in preventing and responding to communicable diseases requires the proper recognition of the contextual factors and challenges, experiences and perception of healthcare providers and decision-makers of the key agencies involved. The authors carried out a qualitative study with conventional content analysis approach about the Arbaeen ceremony, a religious mass gathering in Iran and Iraq and found four main themes: 1- health infrastructure defects in Iraq, 2- poor control of the causative factors of infectious diseases, 3- the low perception of risk in pilgrims, and 4- ineffectiveness of health education. Emerging of All four themes has been verified by the participants’ comments which made results acceptable.

 The method of analysis is appropriately explained which can facilitate the reproducibility. The authors conclude that the main factor in preventing communicable disease is pilgrim-based training long before the ceremony. This conclusion is adequately supported by the results.

I have read this submission. I believe that I have an appropriate level of expertise to confirm that it is of an acceptable scientific standard.
